# Work connectivity behavior after-hours and occupational fatigue in OR nurse-parents: a latent profile analysis and the mediating role of psychological detachment

**DOI:** 10.3389/fpubh.2025.1709488

**Published:** 2026-01-07

**Authors:** Jizhu Qu, Hao Hu, Shijiao Lv, Zelong Cheng, Ranran Zhao, Guangying Wan, Jinbao Mao

**Affiliations:** 1 Operating Room, Shandong Provincial Hospital Affiliated to Shandong First Medical University, Jinan, Shandong, China; 2 School of Nursing, Shandong First Medical University, Taian, Shandong, China

**Keywords:** occupational fatigue, nurse, work connectivity behavior after hours, psychological detachment, Latent Profile Analysis, mediating effect

## Abstract

**Background:**

In China, work connectivity behavior after-hours (WCBA) among operating room nurse who are parents (OR nurse-parents) are associated with increased occupational fatigue, whereas psychological detachment may serve as a potential protective factor. A thorough understanding of the relationship among the three factors is conducive to the management of occupational fatigue.

**Aim:**

Explore the relationship between OR nurse-parents' WCBA and occupational fatigue through Latent Profile Analysis (LPA), and analyze the mediating effect of psychological detachment.

**Methods:**

This study constituted a secondary analysis of cross-sectional data from a prior study involving OR nurse-parents in 15 tertiary hospitals in Shandong Province, China. Inclusion criteria were: (1) registered nurse with >1 year of OR experience; (2) parent of at least one child aged 0–18 years; (3) voluntary informed consent. Exclusion criteria were: (1) temporary staff or interns; (2) on extended leave during the study; (3) major comorbidities. A two-part analytical strategy was used. First, latent profile analysis identified subgroups by WCBA, psychological detachment, and occupational fatigue, with multinomial logistic regression then examining predictors of profile membership. Second, a parallel mediation analysis tested psychological detachment as a mediator between WCBA and occupational fatigue.

**Results:**

Data came from the 724 included OR nurse-parents. LPA revealed a three-profile model: “low WCBA-high psychological detachment-low occupational fatigue group (22%),” “moderate WCBA-moderate psychological detachment-moderate occupational fatigue group (50%),” and “high WCBA-low psychological detachment-high occupational fatigue group (28%).” Multivariate analysis identified working over 10 h daily as a risk factor for the high-risk group. Furthermore, Psychological detachment partially mediated the WCBA- occupational fatigue relationship across all occupational fatigue dimensions, accounting for 17.73%−31.52% of total effects.

**Conclusions:**

Mediation analysis confirmed that psychological detachment partially mediates the relationship between WCBA and occupational fatigue. LPA of WCBA, psychological detachment, and occupational fatigue revealed a three-profile solution among operating room nurse-parents in Shandong Province. A critical finding of LPA is that WCBA moderates the relationship between occupational fatigue and psychological detachment, creating a dual effect: while psychological detachment generally reduces occupational fatigue, its benefit diminishes or reverses under moderate WCBA, likely due to unclear communication expectations. Therefore, effective interventions must address both aspects: managing after-hours connectivity to reduce its intrusion and proactively promoting genuine psychological detachment to mitigate fatigue.

## 1 Introduction

Due to the complexity of nursing work and the specificity of the target group, the prevalence of occupational fatigue among nurses was 71%−94.8% (1, 2). Operating room (OR) nursing work is characterized by high workloads, intensity, frequent emergencies, and irregular schedules, contributing to persistently high levels of occupational fatigue (3). Occupational fatigue not only compromises surgical patient safety but also impairs nurses' emergency response capacity and job satisfaction, increasing turnover intent. Previous studies (4) have shown that nurses with high levels of work-family conflict are more prone to occupational fatigue. OR nurse-parents, in particular, face dual pressures from professional duties and family care, making them especially vulnerable to work–family conflict and thus a high-risk group for occupational fatigue (5). In China, work connectivity behavior after-hours (WCBA, which refers to employees actively or passively using communication devices to handle work-related matters outside of working hours) represents a common yet understudied form of work–family conflict. Although WCBA can improve efficiency (6), it also tends to blur work–life boundaries, prolonging work-related stress and raising occupational fatigue risk among OR nurse-parents (7). Therefore, understanding how WCBA influences occupational fatigue in this population is of considerable importance. Psychological detachment—defined as the mental disengagement from work during off-hours—serves as a key recovery experience that can mitigate occupational fatigue (8, 9). Currently, studies have found that WCBA can reduce psychological detachment (10), increase the frequency of work-family conflict (6), and thereby elevate occupational fatigue (7). However, the mediating role of psychological detachment in the WCBA–occupational fatigue relationship remains unclear, as do the latent profiles defined by these three variables among OR nurse-parents. Given this gap, and considering that workforce shortages often make workload reduction infeasible, identifying recover-focused protective factors like psychological detachment becomes imperative. LPA is a person-centered method that classifies individuals into subgroups based on their response patterns across observed variables, maximizing within-group similarity and between-group difference. This study aims to (1) identify distinct profiles of OR nurse-parents based on work-related communication after hours (WCBA), psychological detachment, and occupational fatigue using latent profile analysis (LPA), and (2) examine the mediating role of psychological detachment in the WCBA-fatigue relationship. By revealing the characteristics of these subgroups, the findings will assist nursing administrators in implementing targeted, minimal-intervention strategies that simultaneously address all three core dimensions—WCBA, psychological detachment, and occupational fatigue—thereby achieving optimal outcomes. The verification of the mediation effect will provide theoretical support for managing WCBA-induced occupational fatigue, ultimately contributing to evidence-based intervention development for this specialized workforce. We hypothesized that: (H1) distinct latent profiles of OR nurse-parents could be identified based on their levels of WCBA, psychological detachment, and occupational fatigue; and (H2) psychological detachment would partially mediate the effect of WCBA on occupational fatigue.

## 2 Methods

According to the “EQUATOR Reporting Guideline Decision Tree,” Using “Latent Class Analysis: Sample Results Report” as our reporting guidelines (11).

### 2.1 Study population and sampling

This cross-sectional study was conducted between October and November 2024 among OR nurses in Shandong Province, China. To ensure a homogeneous study population regarding parenting-related lifestyle factors, we focused on OR nurse-parents with children aged 0–18 years. Participants were recruited using a convenience sampling method from 15 tertiary A-level general hospitals. Specifically, with the coordination of the nursing department in each hospital, the questionnaire link was distributed to the primary OR work group chat (e.g., a WeChat group), and all nurses in these groups who met the following criteria were invited to participate. Inclusion criteria were: (1) registered nurse with >1 year of OR experience; (2) parent of at least one child aged 0–18 years; (3) voluntary informed consent. Exclusion criteria were: (1) temporary staff or interns; (2) on extended leave during the study; (3) major comorbidities. An a priori Monte Carlo power analysis for a simple mediation model was conducted using pilot data from the original study (12). The analysis was configured with the objective to determine the necessary sample size for a target power of 0.8. Parameters were set as follows: maximum sample size was constrained to 724; number of Monte Carlo replications was 1,000; confidence level was 95%; and other settings (e.g., random seed = 1234) were kept at their software defaults. The results indicated that the required sample sizes for the three subscales of variable Y were 443, 200, and 540, respectively. As the final sample size of 724 exceeds the maximum requirement of 540, the study is deemed to be sufficiently powered. The final sample of 724 participants met this requirement.

### 2.2 Data collection and quality control

As a secondary analysis, this study utilized data from an anonymous online survey administered via the www.wjx.cnplatform. Following a pilot study (*n* = 64) to assess feasibility, the formal survey link was distributed to OR work group chats with nursing department coordination. The data quality was ensured through a multi-stage verification process. Invalid responses were flagged for exclusion based on three primary criteria: (1) an unrealistically short completion time (average < 2 seconds per item; (2) patterned responses, detected via a lack of internal consistency with reverse-phrased items embedded in the scales; and (3) implausible or contradictory information, such as reporting fewer total children than children under 18, or a work history in the operating room that exceeded the respondent's age. Subsequently, 724 questionnaires were deemed valid and constituted the final sample, resulting in a valid response rate of 89.35% (13). The study protocol was reviewed and approved by the Shandong Provincial Hospital Ethics Committee for Biomedical Research Involving Human Subjects (SWYX: NO.2024-571). Electronic informed consent was obtained from all participants before they proceeded to the questionnaire.

### 2.3 Instruments

#### 2.3.1 Demographic questionnaire

Design a general information survey form to collect demographic and sociological information about the survey subjects, including gender, age, marital status, number of children, job title, responsibilities, department environment, etc.

#### 2.3.2 Occupational Fatigue Exhaustion Recovery Scale (OFER)

The Chinese version of the Occupational Fatigue Exhaustion Recovery Scale, originally developed by Winwood et al. (14) and cross-culturally adapted by Fang et al. (15), was employed in this study. This instrument consists of 15 items across three subscales: Chronic Fatigue, Acute Fatigue, and Inter-shift Recovery. Responses were recorded on a 7-point Likert scale (0 =“strongly disagree” to 6 =“strongly agree”). Subscale scores were calculated as (sum of item scores/30) × 100. Higher scores on fatigue subscales indicated greater fatigue severity, whereas higher Inter-shift Recovery scores denoted better recovery between shifts. The scale has undergone local validation (16). In our study, Cronbach's α for the three subscales were 0.891, 0.909, and 0.967, respectively.

#### 2.3.3 Psychological detachment

Psychological detachment was assessed using the 4-item Psychological Detachment Subscale from the Recovery Experience Questionnaire (REQ) developed by Sonnentag and Frit (17). Responses were measured on a 5-point Likert scale (1 = “strongly disagree” to 5 = “strongly agree”), with higher scores indicating greater psychological detachment. The scale has undergone local validation (18) and its reliability was further confirmed in our sample with a Cronbach's α of 0.897.

#### 2.3.4 Work connectivity behavior after-hours

This study employed the work connectivity behavior after-hours scale developed by Ma et al. (19). Responses were measured on a 5-point Likert scale ranging from 1 (“never”) to 5 (“always”) with higher scores indicating more frequent work connectivity during non-work hours. The scale has undergone local validation (20), and its reliability was further confirmed in our sample with a Cronbach's α of 0.840.

### 2.4 Statistical analysis

Data analysis was performed using SPSS 27.0 and Mplus 8.0. Descriptive statistics are presented as mean ± standard deviation. LPA was conducted in Mplus 8.0 to identify distinct subgroups of OR nurse-parents based on the manifest variables of work connectivity behavior after-hours, occupational fatigue, and psychological detachment. Model selection was based on a combination of statistical fit indices, model interpretability, and parsimony. Specifically, we considered the Akaike Information Criterion (AIC), Bayesian Information Criterion (BIC), and sample-size-adjusted BIC (aBIC), where lower values indicate better model fit. Classification accuracy was evaluated using entropy, with values closer to 1.00 reflecting higher precision. Improvement in profile solution fit was assessed using the Lo-Mendell-Rubin (LMR) test and the Bootstrap Likelihood Ratio Test (BLRT), with a significant *p-value* (< 0.05) supporting the k-profile model over a model with one fewer profile. Once the optimal profile solution was determined, differences in demographic characteristics across the profiles were examined using chi-square tests or analyses of variance, with a Bonferroni correction for multiple comparisons (corrected *p* < 0.00147, based on 34 tests). The mediating effect of psychological detachment in the relationship between work connectivity behavior after-hours and occupational fatigue was tested using Model 4 in the SPSS macro PROCESS (v4.1) developed by Hayes. The significance of the indirect effect was assessed using a bias-corrected bootstrap method with 5,000 resamples; a 95% confidence interval not containing zero indicated a significant mediation effect. A *p-value* < 0.05 was considered statistically significant.

## 3 Results

### 3.1 Descriptive statistics of key variables

The descriptive statistics of key variables is explained in Table 1.

**Table 1:** OR nurse-parents' psychological detachment, WCBA, occupational fatigue scores (*N* = 724).

**Variable**	***x̄***	**SD**
Psychological detachment	12.06	3.76
WCBA	9.98	2.20
Chronic fatigue	41.90	24.06
Acute fatigue	54.42	23.80
Inter-shift recovery	55.11	19.97

### 3.2 Latent profile analysis identifies distinct fatigue-recovery profiles

**Figure 1:**
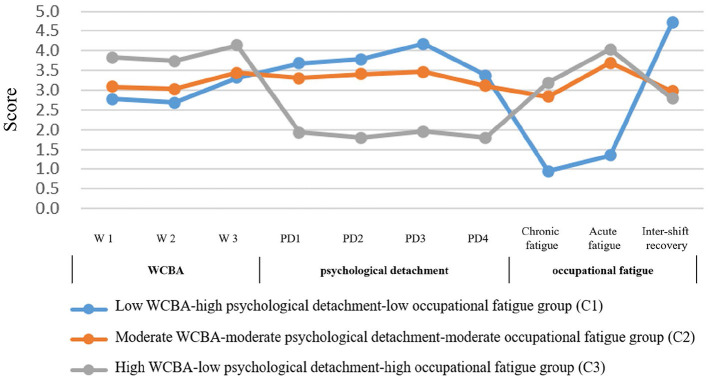
Three- profile of WCBA, psychological detachment, and occupational fatigue among OR nurse-parents. To avoid excessive variation in the vertical axis, the occupational fatigue score is taken as the average score of the questions on the original scale.

We used a three-step LPA to examine patterns among WCBA, occupational fatigue, and psychological detachment (Appendices I–VI). Initially, we estimated models with 1 to 6 profiles using WCBA as the sole indicator. The observation that psychological detachment and occupational fatigue also differed significantly across these preliminary subgroups prompted us to consider introducing additional manifest variables in subsequent LPA analyses. Next, we repeated the LPA incorporating occupational fatigue as a second variable, which again revealed significant between-profile differences in psychological detachment. Finally, a comprehensive model including all three variables was estimated. Based on a comprehensive evaluation of model fit, interpretability, and parsimony, the three-profile solution was selected as the optimal model (Table 2). Although the four-profile model showed a slightly better statistical fit, the three-profile solution demonstrated a similar fit while offering superior conceptual clarity and practical utility for clinical application. Furthermore, the latent class model demonstrated high classification accuracy, with average posterior probabilities for the most likely class ranging from 92.9% to 95.8% (Table 3), indicating excellent distinction between the profiles. The three-profile identified in the final model were labeled as follows: “low WCBA-high psychological detachment-low occupational fatigue group (22%),” “moderate WCBA-moderate psychological detachment-moderate occupational fatigue group (50%),” and “high WCBA-low psychological detachment-high occupational fatigue group (28%).” Multiple comparisons showed that all variables differed significantly across these profiles (Figure 1, Appendix VII).

**Table 2:** WCBA, psychological detachment, and occupational fatigue Latent Profile Analysis model fit indicators for OR nurse-parents.

**Profile**	**AIC**	**BIC**	**aBIC**	**Entropy**	**LMR-adj(P)**	**BLRT(P)**	**Percentage of population**					
							**1**	**2**	**3**	**4**	**5**	**6**
2	32,276.773	32,418.902	32,320.468	0.880	< 0.001	< 0.001	485 (0.67)	239 (0.33)				
**3**	**31,710.134**	**31,902.696**	**31,769.333**	**0.862**	**0.0029**	**< 0.001**	**160 (0.22)**	**364 (0.50)**	**200 (0.28)**			
**4**	**31,336.687**	**31,579.680**	**31,411.390**	**0.864**	**0.0065**	**< 0.001**	**114 (0.16)**	**314 (0.43)**	**120 (0.17)**	**176 (0.24)**		
5	31,090.765	31,384.191	31,180.972	0.850	0.5534	< 0.001	87 (0.12)	152 (0.21)	239 (0.33)	130 (0.18)	116 (0.16)	
6	30,854.205	31,198.064	30,959.917	0.860	0.2377	< 0.001	87 (0.12)	65 (0.09)	217 (0.30)	123 (0.17)	138 (0.19)	94 (0.13)

The bold values mean whereas the four-profile model demonstrated slightly stronger statistical performance, the three-profile model proved to be more interpretable in practice and better suited for informed intervention design. Consequently, the three-profile solution was selected for this study. To fully examine the data, analyses based on the four-profile classification are provided as well.

**Table 3:** Average latent class probabilities.

**Model**	**C1 (%)**	**C2 (%)**	**C3 (%)**
C1	0.929	0.070	0.000
C2	0.038	0.932	0.031
C3	0.001	0.041	0.958

**Figure 2:**
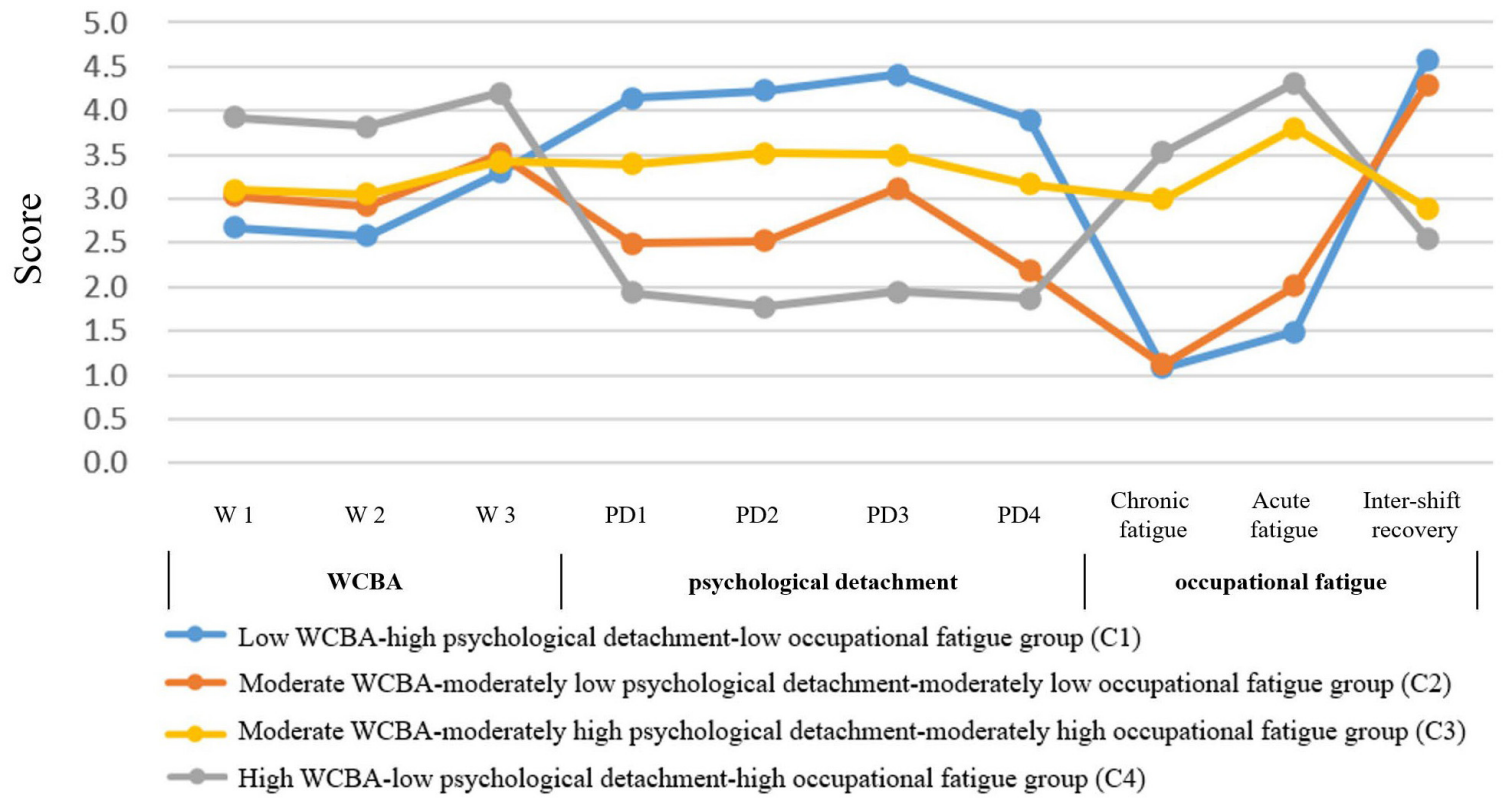
Four-profile of WCBA, psychological detachment, and occupational fatigue among OR nurse-parents. To avoid excessive variation in the vertical axis, the occupational fatigue score is taken as the average score of the questions on the original scale.

We also proceeded to examine the four-profile model and discovered a noteworthy pattern: the association between psychological detachment and occupational fatigue varied across profiles (Figure 2, Appendix VIII). A negative correlation was observed both in the overall three-profile solution (*R* = −0.239, −0.331, 0.249; *P* < 0.001) and in the C1 and C4 subgroups of the four-profile model (*R* = −0.649, −0.757, 0.626; *P* < 0.001). In contrast, this relationship reversed to a positive correlation in the C2 and C3 subgroups of the four-profile model (*R* = 0.405, 0.373, −0.355; *P* < 0.001). These findings suggest that the relationships among these variables are not merely linear but reflect a more complex, underlying structure. Collectively, these results support Hypothesis 1, indicating that operating room nurse-parents in Shandong Province can be meaningfully classified into distinct profiles based on WCBA, occupational fatigue, and psychological detachment.

### 3.3 Multivariate logistic regression analysis of influencing factors

The results of the third latent profile analysis (Profile 3) were treated as outcome indicators. Variables that showed statistical significance after applying a Bonferroni correction for multiple comparisons (*p* < 0.00147) were included as independent variables in the multivariate logistic regression model (Table 4). The analysis revealed that OR nurse-parents reporting lower frequencies of drowsiness and lower consumption of coffee, tea, or functional beverages were more likely to belong to the “low WCBA–high psychological detachment–low occupational fatigue” profile. Conversely, those working more than 10 hours per day were more likely to be classified into the “high WCBA–low psychological detachment–high occupational fatigue” profile. These findings offer practical insights for developing targeted interventions to promote membership in the more adaptive profile (e.g., C1), thereby supporting fatigue mitigation and wellbeing in this population.

**Table 4:** Multifactor analysis of potential profile categories.

**Variable (reference item)**	**Item**	**B**	**Standard Error**	**OR**	**95% C**		***P***
					**Lower limit**	**Upper limit**	
**C2 VS. C1**							
Work-family conflict		0.268	0.046	1.307	1.193	1.431	< 0.001^**^
Drowsiness frequency (D. ≥3 times/week)	B. < 1 time/week	−1.585	0.485	0.205	0.079	0.531	0.001^**^
Position (D. Nurse)	C. Specialist Nurse	1.192	0.442	3.292	1.383	7.836	0.007^**^
Active coping with stressors (D. Often)	A. Never	1.470	0.571	4.349	1.420	13.320	0.010^*^
	B. Occasionally	1.909	0.516	6.746	2.454	18.544	< 0.001^**^
	C. Sometimes	1.004	0.382	2.728	1.289	5.774	0.009^**^
Unhealthy eating due to work (D. Often)	A. Never	−1.359	0.661	0.257	0.070	0.937	0.040^*^
Coffee/tea/energy drink consumption at work (E. Always)	A. Never	−1.975	0.900	0.139	0.024	0.809	0.028^*^
	B. Occasionally	−2.161	0.897	0.115	0.020	0.669	0.016^*^
	C. Sometimes	−2.473	0.925	0.084	0.014	0.517	0.008^**^
	D. Often	−2.713	0.937	0.066	0.011	0.416	0.004^**^
**C3 VS. C1**							
Work-family conflict		0.338	0.049	1.402	1.273	1.544	< 0.001^**^
Family-Work conflict		−0.145	0.048	0.865	0.788	0.950	0.002^**^
Drowsiness frequency (D. ≥3 times/week)	B. < 1 time/week	−2.036	0.568	0.131	0.043	0.398	< 0.001^**^
Position (D. Nurse)	C. Specialist Nurse	1.481	0.499	4.395	1.651	11.698	0.003^**^
Daily working hours (C. >10 h/day)	A. ≤ 8 h/day	−1.761	0.784	0.172	0.037	0.799	0.025^*^
Active coping with stressors (D. Often)	A. Never	1.482	0.641	4.401	1.252	15.472	0.021^*^
	B. Occasionally	1.485	0.580	4.417	1.417	13.767	0.010^*^
Coffee/tea/energy drink consumption at work (E. Always)	A. Never	−2.611	0.938	0.073	0.012	0.461	0.005^**^
	B. Occasionally	−2.441	0.929	0.087	0.014	0.538	0.009^**^
	C. Sometimes	−3.123	0.962	0.044	0.007	0.290	0.001^**^
	D. Often	−2.330	0.961	0.097	0.015	0.639	0.015^*^
Noise levels (D. High)	B. Moderate	−1.570	0.678	0.208	0.055	0.786	0.021^*^
Temperature perception (D. Warm)	C. Comfortable	−2.045	0.864	0.129	0.024	0.704	0.018^*^
**C3 VS. C2**							
Work-family conflict		0.070	0.023	1.073	1.025	1.123	0.003^**^
Family-Work conflict		−0.058	0.020	0.943	0.906	0.982	0.004^**^
Position (D. Nurse)	B. Clinical educator	0.960	0.409	2.612	1.171	5.827	0.019^*^
Daily working hours (C. >10 h/day)	B. 8–10 h/day	−0.565	0.288	0.568	0.323	0.999	0.0497^*^
OR layout ergonomics (C. Favorable)	A. Neutral	0.840	0.368	2.317	1.127	4.763	0.022^*^

^*^P < 0.05, ^**^P < 0.01.

### 3.4 Mediating effect of psychological detachment

**Figure 3:**
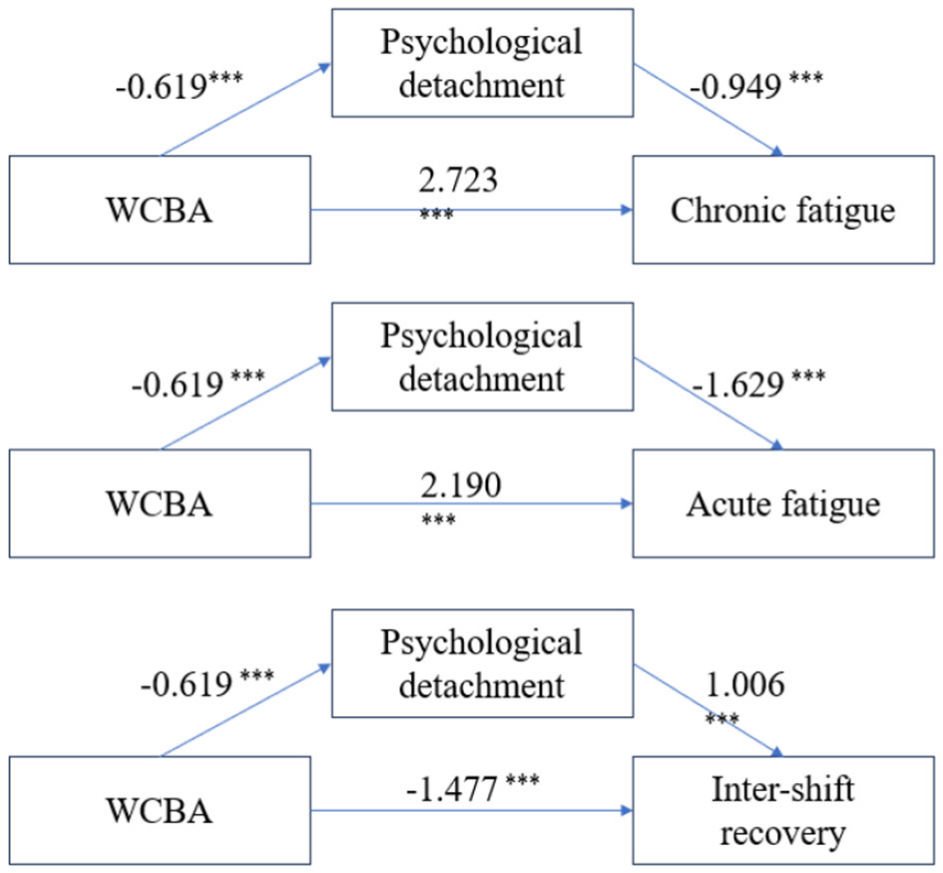
Mediating effect of psychological detachment between WCBA and occupational fatigue. ***P < 0.001.

To examine the mediating role of psychological detachment in the relationship between work-related communication after hours (WCBA) and occupational fatigue, we conducted mediation analysis using Model 4 of the SPSS PROCESS macro (v4.1) with 5,000 bias-corrected bootstrap samples. The results supported a partial mediation model, as both the indirect and direct effects of WCBA on fatigue were statistically significant (95% CIs excluding zero) (see Figure 3, Table 5), thus confirming Hypothesis 2.

**Table 5:** Table showing the decomposition of total effects, direct effects, and mediating effects (*N* = 724).

**WCBA**		**Effect value**	**SE**	**LLCI**	**ULCI**	**Effect size**
**- psychological detachment-**						
Chronic fatigue	Total effect	3.31	0.388	2.549	4.071	
	Direct effect	2.723	0.412	1.945	3.531	82.27%
	Mediating effect	0.587	0.181	0.247	0.961	17.73%
Acute fatigue	Total effect	3.198	0.384	2.443	3.952	
	Direct effect	2.19	0.399	1.406	2.974	68.48%
	Mediating effect	1.008	0.208	0.619	1.434	31.52%
Inter-shift recovery	Total effect	−2.099	0.328	−2.744	−1.455	
	Direct effect	−1.477	0.347	−2.157	−0.796	70.37%
	Mediating effect	−0.623	0.16	−0.953	−0.326	29.68%

Analysis showed that psychological detachment partially mediated the association between WCBA and all three dimensions of occupational fatigue. For chronic fatigue, the indirect effect was 0.587, accounting for 17.73% of the total effect. For acute fatigue, the indirect effect was 1.008, representing 31.52% of the total effect. In the case of inter-shift recovery, WCBA exerted a significant indirect effect of −0.623 through psychological detachment, contributing 29.68% of the total effect. In all cases, the direct effects remained significant, supporting partial mediation.

## 4 Discussion

### 4.1 The dual effect of WCBA on occupational fatigue

Our study indicated a positive correlation between WCBA and occupational fatigue in OR nurse-parents (*R* = 0.303, 0.296, −0.231, *P* < 0.001) (8), supporting the view that WCBA causes off-duty work stress and increases fatigue risk (21). This finding, however, appears to contradict studies suggesting that WCBA is associated with improved efficiency, reduced workload (6) and lower fatigue. This paradox can be explained by differences in job roles. Consistent with previous observations (22), nursing managers generally engage in more WCBA. In our sample, comparative analysis between nurses in managerial (*N* = 79) and non-managerial (*N* = 645) roles confirmed that head nurses showed higher WCBA, lower psychological detachment, yet stronger inter-shift recovery capacity (*P* < 0.001). To interpret these divergent effects, we draw on the empowerment–enslavement paradox (23), a theoretical lens often used to explain the dual nature of communication technologies. For nursing managers with greater autonomy and decision-making latitude, higher WCBA is associated with characteristics of an empowering tool, correlating with improved efficiency, a higher willingness to use ICTs, and better recovery. In contrast, for frontline OR nurse-parents, higher WCBA is correlated with characteristics of a stressor, showing an association with undermined psychological detachment, conflict with a lower ICT willingness, and ultimately, exacerbated fatigue (24, 25). Thus, the relationship between WCBA and fatigue is not uniform but rather bidirectional (26, 27), which may be related to the congruence between WCBA and an individual's ICT-related willingness. Based on this mechanism, we recommend that: (1) nursing administrators could strive to strengthen teamwork and increase employees' sense of autonomy, which may enhance ICT willingness and align it with WCBA demands, potentially contributing to improved efficiency and reduced fatigue (23, 28); and (2) frontline nurses should be encouraged to recognize the dual nature of WCBA, clearly communicate their ICT boundaries, and use technology rationally to maintain personal wellbeing and safety (25, 29, 30).

### 4.2 WCBA as a moderator between psychological detachment and occupational fatigue

Based on the interesting patterns revealed by the four-profile LPA, we further explored the complex relationship between psychological detachment and occupational fatigue. Similar to the findings of Cho et al. (8), psychological detachment was generally negatively correlated with occupational fatigue (*R* = −0.239, −0.331, 0.249; *P* < 0.001), suggesting that disengaging from work-related thoughts after hours is associated with reduced fatigue (9). However, LPA revealed that the relationship between psychological detachment and occupational fatigue is not uniform but is highly conditional on the level of WCBA (Figure 2, Appendices VII–VIII). Specifically, under moderate WCBA levels (subgroups C2 and C3), this relationship reversed, showing a positive correlation between detachment and fatigue (*R* = 0.405, 0.373, −0.355; *P* < 0.001). In contrast, the conventional negative correlation was maintained in both high and low WCBA subgroups (C1 and C4; *R* = −0.649, −0.757, 0.626; *P* < 0.001). This reversal may be explained by the conceptual distinction between detachment state and detachment behavior proposed by Wan et al. (31). Nurses with clearly defined WCBA levels (either high or low) experience predictable communication expectations and lower after-hours electronic communication expectation pressure (AECE) (28), enabling them to achieve a genuine detachment state that supports recovery. In contrast, those under moderate WCBA often face ambiguous communication expectations and higher AECE. In response, they may adopt superficial detachment behaviors—such as muting group notifications—without attaining true psychological recovery. This effort can be counterproductive, as it may be accompanied by underlying anxiety about missing important information, thereby consuming additional psychological resources and ultimately increasing fatigue (31, 32). In summary, our findings are consistent with a model where WCBA moderates the relationship between psychological detachment and fatigue, suggesting that the nature of detachment (whether it is correlated with a beneficial recovery state or a counterproductive behavior) may vary with WCBA levels. These findings suggest that nursing managers should strive to clarify after-hours communication norms, reduce AECE, and help employees achieve true psychological recovery, thereby mitigating fatigue risk (33, 34).

### 4.3 Mediating role and intervention strategies for psychological detachment

According to the Effort-Recovery Model (21), WCBA can be conceptualized as a persistent work demand that is proposed to contribute to occupational fatigue (1). Our cross-sectional findings are consistent with this view, showing a significant association between WCBA and fatigue. Our mediation analysis is consistent with a model where psychological detachment plays a partial mediating role in the relationship between WCBA and fatigue. This exploratory finding suggests that interventions aimed at enhancing detachment might be a promising strategy to mitigate the negative impact associated with WCBA. In the context of the current shortage of operating room nurses and increasing clinical workloads in China, improving psychological detachment represents a practical and targeted intervention strategy. We identified several factors that were associated with psychological detachment and, therefore, could represent potential targets for intervention. At the individual level, poor physical or mental health, extended working hours, and higher professional titles were associated with lower psychological detachment (35), whereas regular exercise, adequate sleep (8), and engagement in offline leisure activities (34) were beneficial. It should be noted that while online leisure may facilitate psychological detachment, it also carries the risk of bedtime procrastination—a challenge often intensified by childcare responsibilities (36). At the organizational level, a supportive social atmosphere and positive leadership practices (32, 37, 38)—particularly when leaders themselves model healthy psychological detachment (39)—were important facilitators. Based on these findings, we recommend that nursing managers focus on the following interventions: (1) Cultivate a supportive team environment and adopt positive leadership styles to encourage psychological detachment among staff (7, 33, 39); (2) Advocate for ergonomic improvements in the workplace to reduce environmental stressors (40); (3) Introduce evidence-based recovery training, such as mindfulness practice (41) or cognitive behavioral therapy (42), to help OR nurse-parents build sustainable recovery skills. At the same time, nurses should be encouraged to prioritize healthy recovery strategies—such as physical activity and offline hobbies (34)—over temporary coping mechanisms like emotional eating or excessive caffeine consumption, which do not support long-term psychological resource restoration.

## 5 Limitations

(1) This study, based on a secondary analysis of cross-sectional data, can only demonstrate correlations between variables rather than establish causal relationships. The discussion section provides a theoretical interpretation of the findings. Furthermore, there may also be confounding factors that affect the correlation. Future longitudinal or experimental studies are needed to verify the causal linkages among the variables.

(2) This study collected data from 15 Grade III Class A general hospitals in Shandong Province, China. The sample was limited to the Shandong region, but data will be collected from a larger region in the future. The study focused on OR nurse-parents, and the universality of the results among other nursing staff needs to be further explored.

(3) Data collection mainly relies on self-administered questionnaires. Although this method is widely used, the results may be affected by recall bias and social desirability bias.

(4) While LPA pointed to a potential moderating role of WCBA, the formal moderation analysis did not yield a statistically significant effect. This discrepancy suggests that the influence of WCBA on the relationship between psychological detachment and occupational fatigue is complex and likely non-linear. Given the limitations of secondary data analysis, in future efforts to further validate causal relationship analysis, in addition to conducting longitudinal or comparative experiments, professional scales should also be used for verification.

(5) A further limitation is that the variables used in the latent profile analysis, derived from scales with different response formats, were not standardized. This may have affected the relative contribution of each variable to the profile derivation.

## 6 Conclusion

Psychological detachment partially mediated the WCBA- occupational fatigue relationship across all occupational fatigue dimensions, accounting for 17.73%−31.52% of total effects. LPA of WCBA, psychological detachment, and occupational fatigue revealed a three-profile solution among operating room nurse-parents in Shandong Province. A critical finding of LPA is that WCBA moderates the relationship between occupational fatigue and psychological detachment, creating a dual effect: while psychological detachment generally reduces occupational fatigue, its benefit diminishes or reverses under moderate WCBA, likely due to unclear communication expectations. Therefore, effective interventions must address both aspects: managing after-hours connectivity to reduce its intrusion and proactively promoting genuine psychological detachment to mitigate fatigue.

## Data Availability

The original contributions presented in the study are included in the article/Supplementary material. Further inquiries can be directed to the corresponding authors.
