# Supplementary material for: Work connectivity behavior after-hours and occupational fatigue in OR nurse-parents: a latent profile analysis and the mediating role of psychological detachment
Source: Front Public Health. 2026 Jan 7;13:1709488. doi: 10.3389/fpubh.2025.1709488 (PMC12819661; doi:10.3389/fpubh.2025.1709488)
Supplement: Supplementary file 1 [file Table_1.docx]

**Appendix**

The first LPA to validate variable correlations and intergroup differences

Appendix Ⅰ. Potential profile analysis model fit indicators for WCBA among OR nurse-parents

| profile | AIC | BIC | аBIC | Entropy | LMR-adj(P) | BLRT(P) | Percentage of population | | | | | |
| --- | --- | --- | --- | --- | --- | --- | --- | --- | --- | --- | --- | --- |
|  |  |  |  |  |  |  | 1 | 2 | 3 | 4 | 5 | 6 |
| 1 | 4567.348 | 4599.442 | 4577.215 |  |  |  | 1.00 |  |  |  |  |  |
| 2 | 4360.078 | 4410.511 | 4375.583 | 0.720 | ＜0.001 | ＜0.001 | 0.65 | 0.35 |  |  |  |  |
| **3** | **4133.541** | **4202.313** | **4154.684** | **0.994** | **0.0113** | **＜0.001** | **0.19** | **0.47** | **0.34** |  |  |  |
| 4 | 2912.736 | 2999.847 | 2939.516 | 1.000 | 0.492 | ＜0.001 | 0.47 | 0.19 | 0.27 | 0.07 |  |  |
| 5 | 2559.979 | 2665.430 | 2592.398 | 0.990 | 0.007 | ＜0.001 | 0.08 | 0.06 | 0.37 | 0.42 | 0.08 |  |
| 6 | 2487.471 | 2611.260 | 2525.527 | 0.991 | 0.006 | ＜0.001 | 0.01 | 0.37 | 0.06 | 0.08 | 0.41 | 0.08 |

| Appendix Ⅱ. Characteristics of potential WCBA categories among OR nurse-parents |
| --- |
| 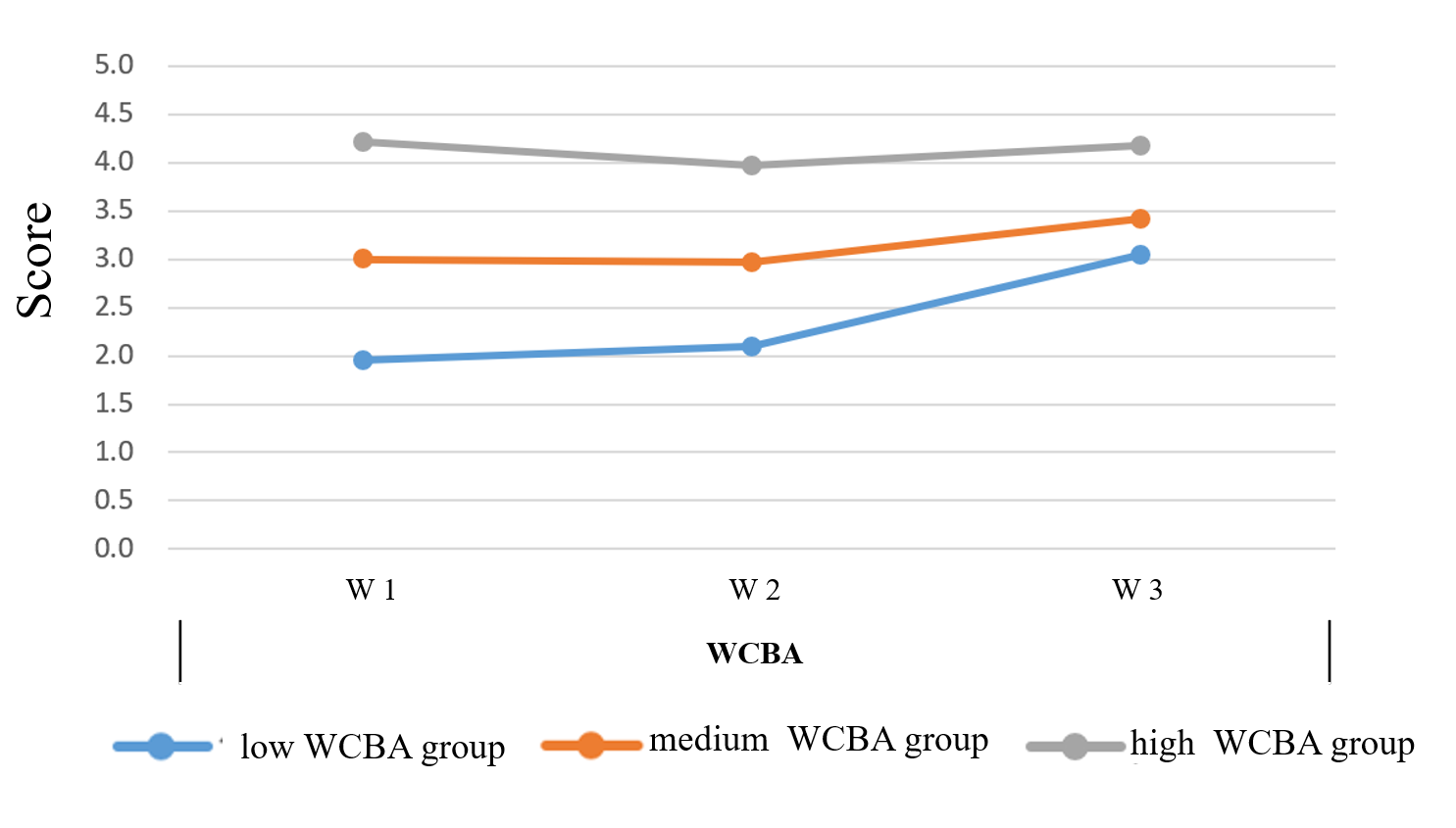 |

| Appendix Ⅲ. Differences in psychological detachment and occupational fatigue scores between the three groups | | | | | |
| --- | --- | --- | --- | --- | --- |
| Variable | low WCBA group | medium WCBA group | high WCBA group | statistic | *P* |
|  | n=135 | n=340 | n=249 |  |  |
| psychological detachment | 14.08±3.3 | 12.29±3.24 | 10.63±4.08 | H=76.08 | ＜0.001 |
| Chronic fatigue | 29.68±21.41 | 40.36±21.93 | 50.63±24.91 | H=65.60 | ＜0.001 |
| Acute fatigue | 43.38±23.44 | 52.91±22.38 | 62.48±23.13 | F=31.99 | ＜0.001 |
| Inter-shift recovery | 63.23±18.33 | 55.8±18.64 | 49.76±21.01 | F=21.46 | ＜0.001 |

The second LPA to validate variable correlations and intergroup differences

Appendix Ⅳ. WCBA and occupational fatigue potential profile analysis model fit indicators for OR nurse-parents

| profile | AIC | BIC | аBIC | Entropy | LMR-adj(P) | BLRT(P) | Percentage of population | | | | | |
| --- | --- | --- | --- | --- | --- | --- | --- | --- | --- | --- | --- | --- |
|  |  |  |  |  |  |  | 1 | 2 | 3 | 4 | 5 | 6 |
| 1 | 12921.404 | 12976.421 | 12938.318 |  |  |  | 1.00 |  |  |  |  |  |
| 2 | 12026.689 | 12113.800 | 12053.470 | 0.790 | ＜0.001 | ＜0.001 | 0.39 | 0.61 |  |  |  |  |
| **3** | **11523.474** | **11642.678** | **11560.121** | **0.990** | **0.007** | **＜0.001** | **0.20** | **0.50** | **0.30** |  |  |  |
| 4 | 11239.053 | 11390.351 | 11285.566 | 0.870 | 0.003 | ＜0.001 | 0.45 | 0.20 | 0.22 | 0.13 |  |  |
| 5 | 11004.049 | 11187.441 | 11060.429 | 0.895 | 0.231 | ＜0.001 | 0.17 | 0.15 | 0.19 | 0.39 | 0.11 |  |
| 6 | 9864.667 | 10080.152 | 9930.914 | 0.932 | 0.012 | ＜0.001 | 0.19 | 0.14 | 0.08 | 0.33 | 0.19 | 0.07 |

Appendix Ⅴ. Three potential profiles of WCBA and occupational fatigue among OR nurse-parents

| 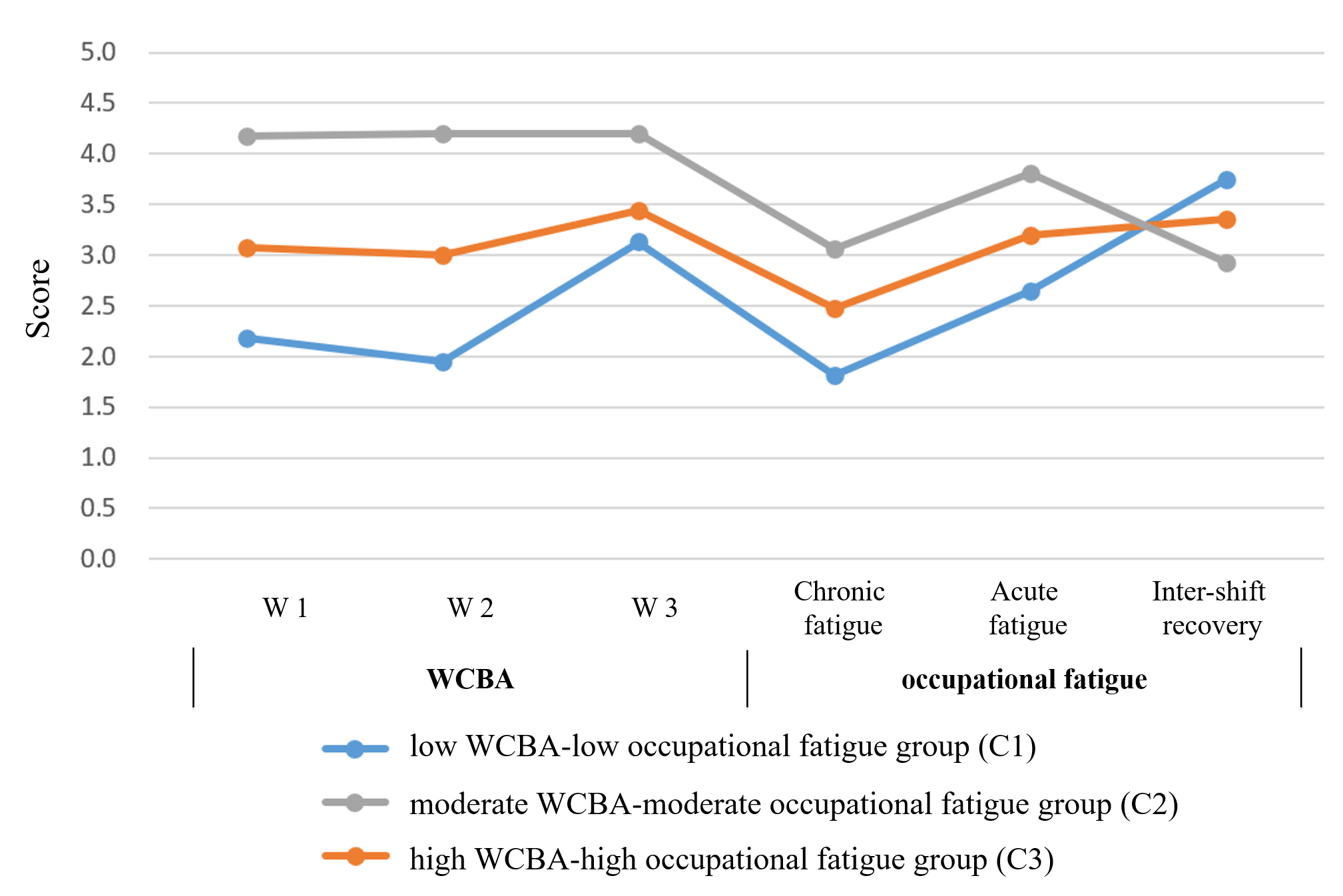 |
| --- |

Appendix Ⅵ. Differences in psychological detachment and occupational fatigue scores between the three groups

| Variable | C1 | C2 | C3 | statistic | multiple comparisons |
| --- | --- | --- | --- | --- | --- |
| WCBA | 7.26±1.3 | 9.52±0.9 | 12.59±1.25 | 564.082** | 1＜2＜3 |
| psychological detachment | 14.03±3.32 | 12.22±3.3 | 10.44±4.07 | 77.978** | 1＞2＞3 |
| Chronic fatigue | 30.27±21.06 | 41.16±22.29 | 51.05±25.21 | 62.343** | 1＜2＜3 |
| Acute fatigue | 44.08±23.47 | 53.23±22.75 | 63.46±22.52 | 59.509** | 1＜2＜3 |
| Inter-shift recovery | 62.36±19.45 | 55.99±18.42 | 48.7±20.92 | 46.265** | 1＞2＞3 |

C1：low WCBA-low occupational fatigue group

C2：moderate WCBA -moderate occupational fatigue group

C3：high WCBA -high occupational fatigue group

** P＜0.001

The third LPA

Appendix Ⅶ. Differences in WCBA, psychological detachment, and occupational fatigue scores among the three groups of subjects

| Variable | C1 | C2 | C3 | statistic | multiple comparisons |
| --- | --- | --- | --- | --- | --- |
| WCBA | 8.78±1.95 | 9.55±1.83 | 11.71±2.01 | 184.252** | 1＜2＜3 |
| psychological detachment | 15±2.96 | 13.28±2.34 | 7.47±1.87 | 436.899** | 1＞2＞3 |
| Chronic fatigue | 15.71±12.37 | 47.28±19.06 | 53.07±24.24 | 261.983** | 1＜2＜3 |
| Acute fatigue | 22.38±14.53 | 61.48±15.49 | 67.22±19.25 | 335.314** | 1＜2＜3 |
| Inter-shift recovery | 78.63±12.96 | 49.52±13.71 | 46.47±19.88 | 279.086** | 1＞2=3 |

C1：Low WCBA -high psychological detachment-low occupational fatigue group

C2：Moderate WCBA -moderate psychological detachment-moderate occupational fatigue group

C3：High WCBA -low psychological detachment-high occupational fatigue group

** P＜0.001

Appendix Ⅷ. Differences in WCBA, psychological detachment, and occupational fatigue scores among the four groups of subjects

| Variable | C1 | C2 | C3 | C4 | statistic | multiple comparisons |
| --- | --- | --- | --- | --- | --- | --- |
| WCBA | 8.55±2.03 | 9.46±1.88 | 9.59±1.8 | 11.94±1.87 | 202.719** | 1＜2=3＜4 |
| psychological detachment | 16.67±2.06 | 10.32±2.07 | 13.58±2.14 | 7.53±1.99 | 522.448** | 1＞3＞2＞4 |
| Chronic fatigue | 18.13±14.07 | 18.72±13.96 | 49.95±17.49 | 58.75±21.1 | 361.605** | 1=2＜3＜4 |
| Acute fatigue | 24.77±16.51 | 33.58±17.97 | 63.33±14.15 | 71.95±16.26 | 395.741** | 1＜2＜3＜4 |
| Inter-shift recovery | 76.32±13.57 | 71.58±15.11 | 48.15±13.3 | 42.56±18.25 | 332.718** | 1=2＞3＞4 |

C1: Low WCBA-high psychological detachment-low occupational fatigue group

C2: Moderate WCBA-moderately low psychological detachment-moderately low occupational fatigue group

C3: Moderate WCBA-moderately high psychological detachment-moderately high occupational fatigue group

C4: High WCBA-low psychological detachment-high occupational fatigue group

** P＜0.001
